# Human Organotypic Cultured Cardiac Slices: New Platform For High Throughput Preclinical Human Trials

**DOI:** 10.1038/srep28798

**Published:** 2016-06-30

**Authors:** C. Kang, Y. Qiao, G. Li, K. Baechle, P. Camelliti, S. Rentschler, I. R. Efimov

**Affiliations:** 1 Washington University in St. Louis, St. Louis MO, USA; 2 The George Washington University, Washington DC, USA; 3 University of Surrey, Guildford, UK; 4 Moscow Institute of Physics and Technology, Moscow, Russia; 5 IHU LIRYC, Site Xavier Arnozan, Avenue du Haut Lévêque, Pessac, France

## Abstract

Translation of novel therapies from bench to bedside is hampered by profound disparities between animal and human genetics and physiology. The ability to test for efficacy and cardiotoxicity in a clinically relevant human model system would enable more rapid therapy development. We have developed a preclinical platform for validation of new therapies in human heart tissue using organotypic slices isolated from donor and end-stage failing hearts. A major advantage of the slices when compared with human iPS-derived cardiomyocytes is that native tissue architecture and extracellular matrix are preserved, thereby allowing investigation of multi-cellular physiology in normal or diseased myocardium. To validate this model, we used optical mapping of transmembrane potential and calcium transients. We found that normal human electrophysiology is preserved in slice preparations when compared with intact hearts, including slices obtained from the region of the sinus node. Physiology is maintained in slices during culture, enabling testing the acute and chronic effects of pharmacological, gene, cell, optogenetic, device, and other therapies. This methodology offers a powerful high-throughput platform for assessing the physiological response of the human heart to disease and novel putative therapies.

## Motivation

Cardiovascular research on the mechanisms of heart failure and sudden death has been conducted primarily in animal models with varying degree of clinical relevance. While animal models are indispensable in advancing basic cardiac biology, their translational potential is being challenged due to a paucity of successful translation from bench to bedside in recent years^1^,^2^. Recently, we explored a growing opportunity to augment applied cardiovascular research using live donor and failing human hearts, which provide important insights into human physiology and pharmacology.

### Significance

Despite significant effort and investment from the cardiovascular research community, current heart failure therapies are primarily based on very old ideas, including β-blockers, diuretics, heart transplantation, pacemakers, defibrillators, and ventricular assist devices. New approaches, including gene and cell therapies and tissue engineering, are yet to prove clinical relevance, while survival rates on a typical drug regimen remain approximately 1 year^3^. The typical heart failure drug regimen includes angiotensin blockers, β-blockers, calcium channel blockers, anticoagulants, antiplatelets, vasodilators, and digitalis, which do little to address the underlying etiology of heart failure^4^. As heart failure burden increases with age, it is very difficult for older patients to keep up with the cost and complexity of these drug regimens^5^. Novel drug development is a slow and expensive process due in a large degree to the lack of reliable high throughput screening technology preceding clinical trials. Limited genetic and physiological relevance of many animal models of heart failure to the human condition also results in very low clinical translation of new therapies^6^.

### Comparison of current models

There are substantial barriers impeding the translation of discoveries made in animal models into humans, including scarcity of clinically relevant *in vitro* human model systems for evaluation of putative therapies. The organotypic tissue slice is a widely used technique to study diverse organ systems, including the brain, kidney, and liver^7^,^8^. We have adopted this technique to human cardiac tissue, providing us with a multicellular model system enabling mechanistic studies of cardiac physiology and gene therapy approaches in a human primary substrate system. There are several unique advantages that the cardiac organotypic slice culture model affords over well established models. First, the lack of multi-cellularity and intercellular contacts make isolated cells unsuitable for investigation of multi-cellular events such as cell-cell communication, cardiac conduction, and arrhythmia. Additionally, the chunk dissociation procedure used to study isolated human cell electrophysiology utilizes an enzymatic digestion step that appears to significantly degrade selected potassium channel function^9^. While iPS cells are very useful for lineage specification and developmental electrophysiology studies^10^,^11^, it remains challenging to differentiate iPS cells into mature, rod-like quiescent adult atrial or ventricular myocytes. Finally, coronary perfused intact tissue preparations (wedge preparations) provide a well established system to study intact tissue-level physiology, however, preparations are large and require coronary perfusion limiting the number of preparations that can be dissected from a human heart^12^,^13^. Furthermore, wedge preparations can only be maintained for several hours, which prevents long-term chronic experimental investigation, including gene expression and proliferative responses. Despite these advantages over currently utilized models, cardiac slices are not designed to replace, but rather, to fill a niche among them. Isolated myocytes remain the only model to study specific ionic currents, iPSC-derived cardiomyocytes can provide patient specific therapy screening, and coronary perfused preparations provide detailed insight into three dimensional conduction and arrhythmia properties within tissues (Supplemental Table 1).

### Current state of cardiovascular slice

Several recent publications have indicated that the cardiac slice preparation has an enormous untapped potential to augment and bridge the currently utilized isolated primary cells and coronary perfused heart preparations^6^,^14^,^15^. Organotypic slices preserve the native tissue environment, allowing normal contacts with extracellular matrix and intercellular communication, which facilitates the maintenance of the differentiated adult cardiomyocyte phenotype^16^,^17^,^18^. Several publications demonstrated that slices from rabbit, guinea pigs, mouse, and canine hearts exhibit similar electrophysiology to the intact heart via multi-electrode array measurements^15^,^17^. Additionally, acute delivery of drugs including the hERG-type potassium channel blocker, E-4031, and the slow delayed rectifier potassium channel blocker, chromanol 293B, have exhibited a comparable effect in slice versus coronary perfused preparations^17^. Furthermore, slices have previously shown robust survivability in culture. However, significant mechanical and electrophysiological remodeling was noted during long-term culture in these studies^16^.

Most prior electrophysiology studies relied on multi-electrode arrays, which are limited by the low electrode density (8 × 8 to 16 × 16 electrodes) and the inability to capture action potential morphology^17^,^18^,^19^. In contrast, optical mapping has a far higher resolution of 100 × 100 or more pixels and the ability to map action potentials, calcium transients, mitochondrial inner member potential, etc.^20^ In this study, we aimed to develop adult human cardiac slices from atria and ventricles as a preclinical screening platform for novel drug, gene, or other types of therapies. Utilizing the optical mapping technique, we provide a high-resolution electrophysiological characterization of adult human atrial and ventricular slices subjected to drugs and viral gene delivery vectors, in fresh slices and following 24–96 hours in culture.

## Methods

### Human tissue acquisition

Adult human hearts were procured either as failing human hearts from Barnes-Jewish Hospital (St. Louis, MO) or Inova Fairfax Hospital (Fairfax, VA), and as donor human hearts from Mid-America Transplant Services (St. Louis, MO) or Washington Regional Transplant Community (Washington, DC). Experimental protocols were approved by both Washington University and the George Washington University Institutional Review Boards (IRBs). Informed donor consents were obtained for all tissue used in this study. Methods described in this section were performed in accordance with all human research guidelines. Explanted hearts were cardioplegically arrested via high potassium solution (in mmol/L: NaCl 110, CaCl_2_ 1.2, KCl 16, MgCl_2_ 16, NaHCO_3_ 10) and were cooled to +4 °C in the operating room following aortic cross-clamp. Failing hearts were perfused through both coronaries, while donor hearts were perfused systemically prior to removal from the chest. The heart was maintained at this low temperature to preserve tissue during the 15–20 minute transportation time from the operating room to the research laboratory.

### Slice preparation

Upon arrival, an approximately 1cm by 1cm cube of left ventricular tissue or right atrial tissue was cut in cardioplegic solution. Left ventricular tissue was taken from a region close to the left anterior descending artery and circumflex artery. Atrial tissue was taken from the crista terminalis below the sinoatrial node region. Premade 4% agarose gel cooled at 4 °C was glued on to the tissue holder of a high precision vibrating microtome (7000 smz-2, Campden Instruments Ltd. UK). The tissue block was then mounted with the endocardium up on the agarose. Agarose did not prevent tissue superfusion. Slices were cut tangential to the endocardium. The cutting chamber was filled with cold (4 °C) oxygenated (100% O_2_) modified Tyrode’s solution with excitation contraction uncoupler 2,3-butanedione (Tyrode’s cutting solution, in mM: NaCl 140; KCl 6; glucose 10; HEPES 10; MgCl_2_ 1; CaCl_2_ 1.8; BDM 10; pH 7.4)^17^. The outer chamber is constantly refilled with ice to maintain constant 4 °C in the cutting chamber.

The microtome was pre-set to 380 μm cutting thickness, 0.02–0.03 mm/s advance speed, 2 mm horizontal vibration amplitude, and 80 Hz vibration frequency. The microtome’s z-axis vibration was also calibrated prior to each experiment with the ceramic cutting blade to <0.5 μm, which limited damage to a single layer of cardiomyocytes during the cutting procedure. After each slice was cut, they were transferred immediately to oxygenated (100% O_2_) washout solution at 20 °C (Tyrode’s recovery solution, in mM: NaCl 140; KCl 4.5; glucose 10; HEPES 10; MgCl_2_ 1; CaCl_2_ 1.8; 1% Penicillin-Streptomycin; pH 7.4). Slices were placed in 100 μm nylon mesh cell strainers in 6 well plates with a bottom drilled through for oxygenation. A meshed washer was placed on top of the slice to prevent the tissue from curling. The tissue slices were kept in the washing solution for 20 minutes to washout the BDM and warm the tissue gradually to room temperature. The tissue could stay in washout solution at room temperature for up to 12 hours for acute electrophysiology studies.

### Acute optical mapping for electrophysiology and drug response

Ventricular slices were electrophysiologically studied with the optical mapping technique as published previously^20^. All optical mapping studies for both fresh and cultured slices were performed in the washout solution (Tyrode’s recovery solution) described above at 37 °C. To eliminate transmural and spatial heterogeneity in the ventricle, we used slices from the sub-endocardium on the anterior of the left ventricle for both validation and chronic drug studies. Optical data was acquired using an Ultima-L CMOS camera system (SciMedia). Transmembrane potential (V_m_) was measured using di-4-ANEPPS and RH237 (Life Technologies). Intracellular calcium [Ca^2+^] was measured using Rhod-2AM (Life Technologies). The dye were loaded very slowly on top of the tissue and allowed to incubate for up to 30 minutes. The excitation-contraction uncoupler, blebbistatin (Cayman Chemical), was also loaded in a similar manner. Using a 50 mm Nikon lens, a 1 × 1 cm field of view was projected to a 100 × 100 pixel CMOS sensor. This ensured that nearly the entire field of view was taken up by tissue to maximize resolution. Optical action potential was captured every 10 minutes during the dye incubation period to ensure optimal signal quality.

Acute electrophysiology measurement included optical mapping of V_m_ and [Ca^2+^] during a restitution pacing protocol. These parameters included action potential duration (APD), activation sequence, calcium transient (CaT) duration and morphology, conduction velocity (CV), restitution properties, and effective refractory period (ERP). Basic drug response was examined using saturated dosage of isoproterenol (100 nM). All ventricular slices did not exhibit automaticity and were paced with custom designed platinum rod field/array pacing chamber. The pacing stimulus was set at twice the amplitude of the pacing threshold and 2 ms pulse duration. Some atrial slices obtained near the *crista terminalis* exhibited automaticity, but they were paced to acquire standardized action potential or calcium transient characteristics.

### Culture procedure, materials, and validation

Slices were cultured in medium 199 (M4530, Sigma Aldrich) supplemented with 2% penicillin-streptomycin, 1 X ITS (Insulin, Transferrin, Selenium) liquid media supplement, and 10 mM 2,3-butanedione monoxime. Slices were individually washed in sterile PBS solution 6 times before placing in culture. Sterile forceps were used to handle slices at all time. Slices were cultured at a liquid-air interface using porous transwell inserts (PICM0RG0, Millipore, USA). Inserts were placed in 6-well culture plates with 1.1 ml culturing medium and placed in a 37 °C incubator with humidified air with 5% CO_2_. The culture medium was changed daily. After a 24 hour culture period, slices’ electrophysiology was measured with optical mapping to examine potential functional changes occurred during culture. APD, action potential morphology, and restitution properties were examined following the same protocol as under acute conditions. A newer iteration of this process simplifies this procedure by removing the PBS wash step in favor of autoclaving.

### Chronic drug model

Utilizing cultured slices and an adult dosage of phenylephrine, a widely used selective α_1_-adrenergic receptor agonist, we developed a model to study chronic drug effects in human cardiac slices. Slice electrophysiology was first acutely measured using optical mapping after application of 3 μM phenylephrine, which was chosen for this study because it is an average of the EC_50_ values found in the literature^21^,^22^,^23^. Then acute slices were placed in either control culture with standard culture medium described above or drug medium including 3 μM phenylephrine. Slices were cultured in a tissue incubator for 24 hours. Control cultured slices were measured before and after phenylephrine application similar to acute slices, slices cultured in drug media were measured separately. We also utilized custom made platinum tipped electrodes for point simulation of slices. This allowed us to more accurately characterize conduction velocity. Only transverse conduction velocity could be measured accurately due to the small size of the tissue.

### Adenoviral transduction and expression of green fluorescent protein

To increase depth of viral tissue penetration and achieve greater viral transduction efficiency, slices were treated with collagenase after they were placed on transwell inserts. 15 μL of Type 2 collagenase (Worthington Biochem) reconstituted at 250 units/mL in slice culture medium was pipetted onto each slice and placed in a tissue incubator for 20 minutes. The slices were subsequently washed in the wells after the incubation period, followed by addition of 1.1 mL of the culture medium to the Transwell. Ad5-GFP (total of 1.11e10 viral particles, 2.82e12 v.p./mL) encoding *eGFP* and *luciferase* driven by the cytomegalovirus promoter was diluted in a total volume of 12 uL PBS, pipetted onto each slice to cover completely, and incubated on the slice for 2–12 hours.

### Histology and Immunohistochemistry

Post experiment, slices are preserved in O.C.T. compound (Tissue-Tek), frozen and sectioned transmurally. Immunohistochemistry was performed using antibodies directed against Connexin 43 (Life Technologies 710700, 1:100 dilution) and α-actinin (Sigma A2172 MFCD00164521, 1:100 dilution). Masson’s trichrome staining was performed as described previously^24^.

### Data analysis methods

A custom data analysis program, Rhythm, developed in our laboratory for optical mapping data was used to analyze V_m_ and [Ca^2+^]^25^. Action potential and calcium transient optical signals were low-pass filtered at 100–150 Hz, spatially averaged at 3 × 3 pixels, normalized from 0 to 1, and fluorescent drift was removed with a first-order fitted curve, if needed. All sample averages are presented with standard error of the mean (S.E.M), which appears as error in figures with bar graphs comparing means. Standard deviation (S.D.) is used in restitution curves. Comparative statistic is performed using one-way ANOVA with post-hoc Tukey honestly significant difference (HSD) test for multiple comparisons unless otherwise noted.

The activation map was plotted via calculating activation time points at 50% of action potential upstroke amplitude. Action potential and calcium transient duration was calculated at 80% repolarization/relaxation. When slices were field-paced, conduction velocity could not be quantified via traditional longitudinal and transverse direction. During point pacing we calculated conduction velocity using a semi-automated Matlab function ORCA^26^. Effective refractory period was measured using S1-S1 pacing protocol until 1:1 capture was lost. Action potential and calcium transient were recorded at pacing cycle lengths from 2000 ms to 200 ms.

## Results

**Figure 1:**
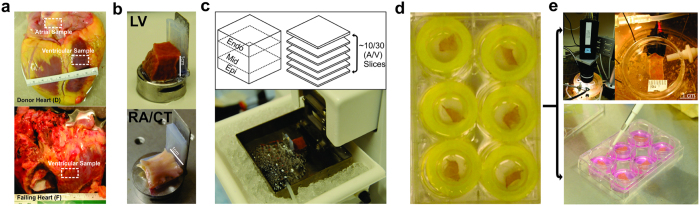
Organotypic Human Cardiac Slice Methodology. Human atrial and ventricular slices were procured from both (**a**) non-failing donor (D) and end stage failing (F) hearts. (**b**) 1cm × 1cm samples from either the left ventricle or the atria were prepared for sectioning. (**c**) Up to 30 sections were made using a vibratome at a cutting rate of 0.02–0.04 mm/s in ice-cold oxygenated modified Tyrode’s solution. (**d**) Slices were conditioned at room temperature for 20 minutes before use for either (**e**) electrophysiology optical mapping, and/or culture and viral transduction.

Cardiac slice preparations have been previously utilized in small human biopsies and animal hearts^16^,^17^, however, systematic slicing protocols have yet to be adapted for the explanted human heart. In collaboration with heart transplantation programs and organ procurement organizations in St. Louis, MO and Washington, DC, we established a robust pipeline for applied human heart research^27^. Hearts were procured from either end stage heart failure patients at the time of transplantation, or from non-failing donors that were not acceptable for transplantation. Figure 1 illustrates the methodology of slicing human atrial and ventricular tissue. In this example, atrial slices were taken from the *crista terminalis* region bordering the sinoatrial (SA) node, while ventricular slices were taken from the left or right ventricular free wall. The block of tissue is placed on a tissue holder with a back support, which prevents the tissue from sliding during slicing. Slices were cut tangentially to the epicardium while paying careful attention to the layer (i.e. subendocardial, subepicardial), because cellular electrophysiology is known to change gradually from the epicardium to the endocardium^28^. After slices were placed in Tyrode’s recovery solution, acute recordings were performed in different tissue layers to determine baseline electrophysiology.

### Quantification of electrophysiology utilizing optical mapping

Multi-electrode arrays are a popular and easy to use tool for assessment of cardiac and neural electrophysiology. In contrast to multi-electrode array, optical mapping of cardiac slices has been shown to faithfully reproduce morphology of action potentials in animal cardiac slices^20^. Here, we apply multiparametric optical mapping for the first time in human atrial and ventricular slices both acutely and after culture.

**Figure 2:**
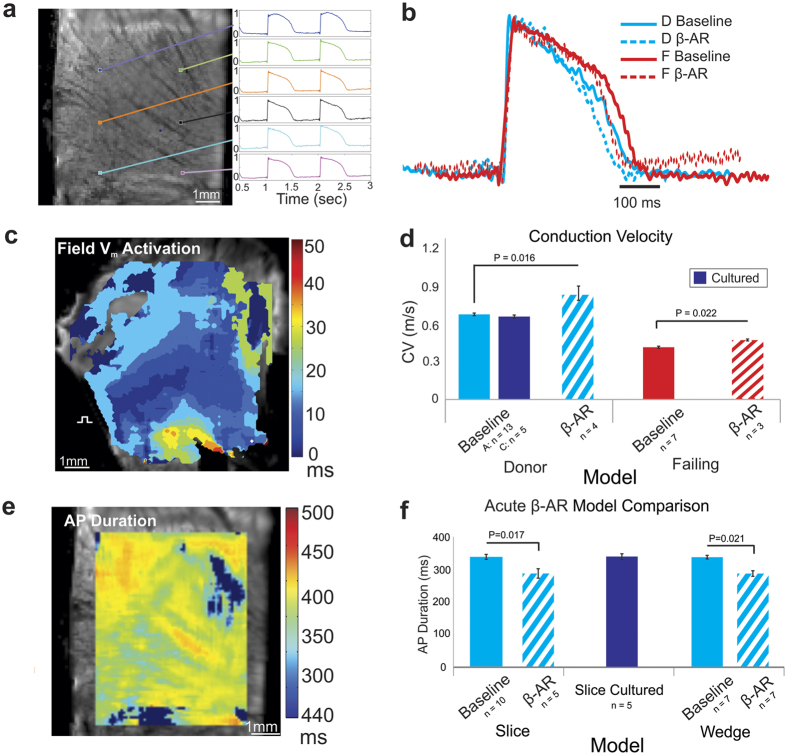
Electrophysiological Properties of Human Ventricular Slices. (**a**) Bright field image of a ventricular slice with sample optical signals from various regions illustrating high signal to noise ratio. (**b**) Action potentials (AP) at 1 Hz pacing frequency (1000 ms pacing cycle length) of failing versus donor slices at baseline and β-adrenergic receptor (AR) stimulation conditions. (**c**) Activation map from a slice during electrical field stimulation demonstrating anisotropic conduction. (**d**) Quantitative summary of conduction velocity showing increased velocity during β-AR stimulation in both donor and failing slices, while culture process does not significantly alter conduction. (**e**) Map of AP duration (APD_80_) at 1 Hz pacing. (**f**) Comparison of APD_80_ at 1 Hz pacing frequency in fresh slices, slices cultured for 24 hr, and in the well-established human coronary perfused ventricle preparation (wedge) model. No significant difference is observed among models. Furthermore, β-AR stimulation decreases APD_80_ to a similar degree in slices and wedge preparations, demonstrating viability of the ventricular slice model for use in drug testing.

Figure 2 summarizes a broad electrophysiological assessment of ventricular slices illustrating the robustness of this model for applied human research. Slices from the left ventricular free wall from either donor or failing hearts were sectioned. Electrophysiology was mapped with high spatial and temporal resolution either acutely, or after 24 hours of culture, with or without pharmacological treatment (Fig. 2a,b). Activation was measured at 50% of depolarization, and conduction velocity (CV) was measured from the activation sequence. AP duration (APD) was calculated by measuring the time between activation and repolarization completed by 80%. Figure 2c presents a typical activation map during field stimulation. To determine whether slices preserve drug responsiveness, we employed well-characterized drugs. A typical response to β-adrenergic stimulation is increased CV and shortened APD^29^,^30^. Donor left ventricular slices paced at 1 Hz (60 beats per minute) exhibited a CV of 0.67 ± 0.008 m/s (±S.E.M) at baseline (n = 13), with an increased CV of 0.84 ± 0.02 m/s during β-adrenergic stimulation with isoproterenol (n = 4) (Fig. 2d, p = 0.016, *one-way ANOVA with post-hoc Tukey HSD*). As expected, heart failure caused slowing of CV at baseline to 0.47 ± 0.003 m/s (n = 7), but isoproterenol increased CV to 0.54 ± 0.003 m/s (Fig. 2d, n = 3, p = 0.022, *two tailed t-test assuming unequal variances*). Importantly, 24 hour culture did not affect CV in donor slices (0.66 ± 0.002 m/s, n = 5) versus baseline (Fig. 2d). Culture degraded the electrical properties of failing slices, therefore we did not include them in subsequent culture studies. This degradation could result from the fact that the end stage failing hearts exhibit a significantly greater degree of fibrosis, depraved metabolic state, and structural degradation at baseline, which are compounded during culture, leading to drastically decreased survival.

Figure 2e shows a typical map of slice ventricular APD, demonstrating a similar baseline APD in slices (337 ± 7 ms, n = 10) when compared with the “gold standard” coronary perfused left ventricular wedge preparations (322 ± 5 ms, n = 7) (Fig. 2f). As expected, β-adrenergic stimulation of slices resulted in a significant decrease of APD from baseline to 284 ± 9 ms (n = 5) (p = 0.017, *one-way ANOVA with post-hoc Tukey HSD*), similar to the decrease from baseline to 290 ± 6 ms (n = 7) seen in wedge preparations (p = 0.021, Fig. 2f). Importantly, APD was also maintained after 24 hours in culture (337 ± 7 ms, n = 10) when compared with acute slices (339 ± 13 ms, n = 5, p = 0.99), which validates this model for use in chronic studies of repolarization (QT toxicity) (Fig. 2f).

### Multi-parametric optical mapping

**Figure 3:**
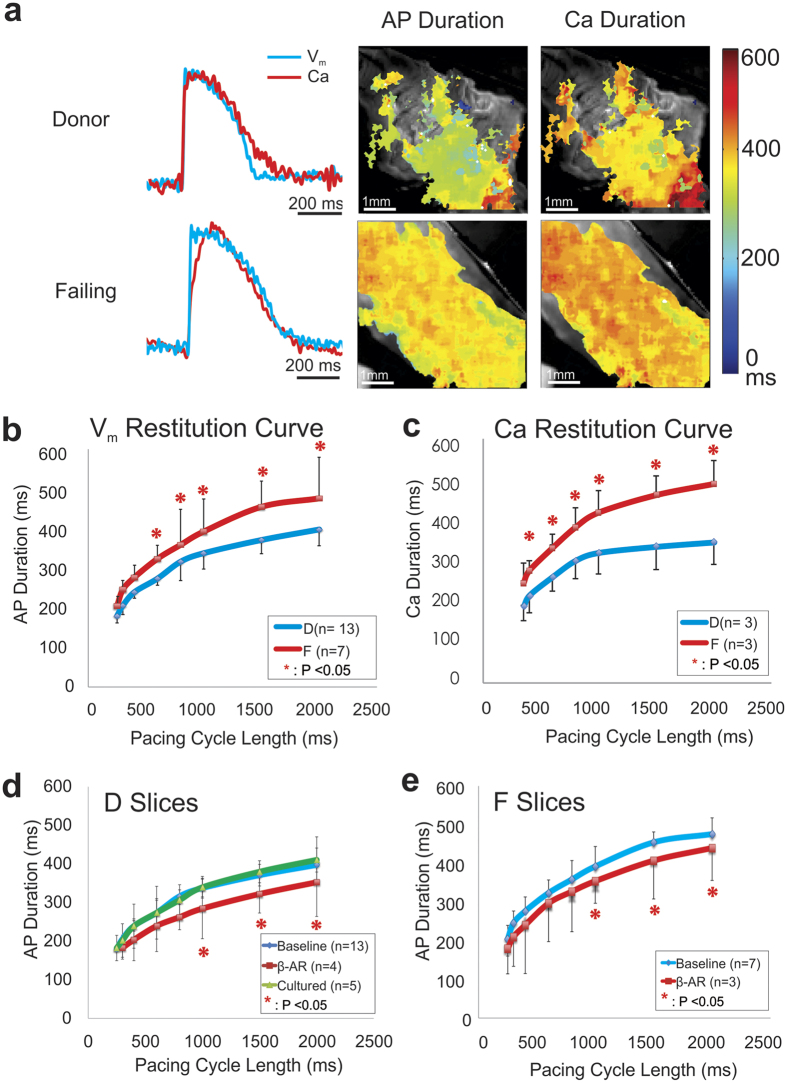
Comparison of Action Potentials and Calcium Handling in Human Ventricular Slices. Action potentials (AP) and calcium transients (CaT) were recorded from the same slices stained with voltage- and calcium-sensitive dyes. (**a**) Comparison of local AP and CaT optical signal obtained from the respective donor and failing slices. (**b**) The baseline APD restitution curve is higher in failing versus donor slices, as expected. (**c**) CaT restitution curves demonstrate a similar difference in failing slice when compared with donor slices to that observed in AP. (**d**) Baseline restitution curve of donor slices is similar to that measured previously in coronary perfused wedge preparations. Acute β-adrenergic stimulation significantly lowers the restitution curve, while culturing slices for 24 hrs did not alter the restitution curve. (**e**) Slices obtained from failing hearts also demonstrate a response to acute β-adrenergic stimulation.

We also conducted multi-parametric optical mapping in cardiac slices by combining transmembrane potential (V_m_) sensitive dye and cytosolic calcium (Ca) sensitive dye (Fig. 3a). Using optical AP and calcium transient (CaT) recordings, we measured restitution properties (waveform duration versus stimulation cycle length) (Fig. 3b,c). As expected, a significant difference was observed in donor versus failing CaT duration (321 ± 18 ms vs 418 ± 20 ms at 1000 ms cycle length; n = 3, p = 0.0062, *two tailed t-test assuming unequal variances*). APD and CaT duration were significantly different between donor and failing hearts at most pacing cycle lengths (Fig. 3b,c, *one-way ANOVA with post-hoc Tukey HSD for each cycle length*) similar to previous reports utilizing wedge preparations^13^,^30^, providing further validation of our tissue slice model. As expected, β-adrenergic stimulation shifted the restitution curve downwards in both donor and failing slices. Culture did not change restitution properties (Fig. 3d,e).

### Characterization of atrial muscle and SA nodal electrophysiology

**Figure 4:**
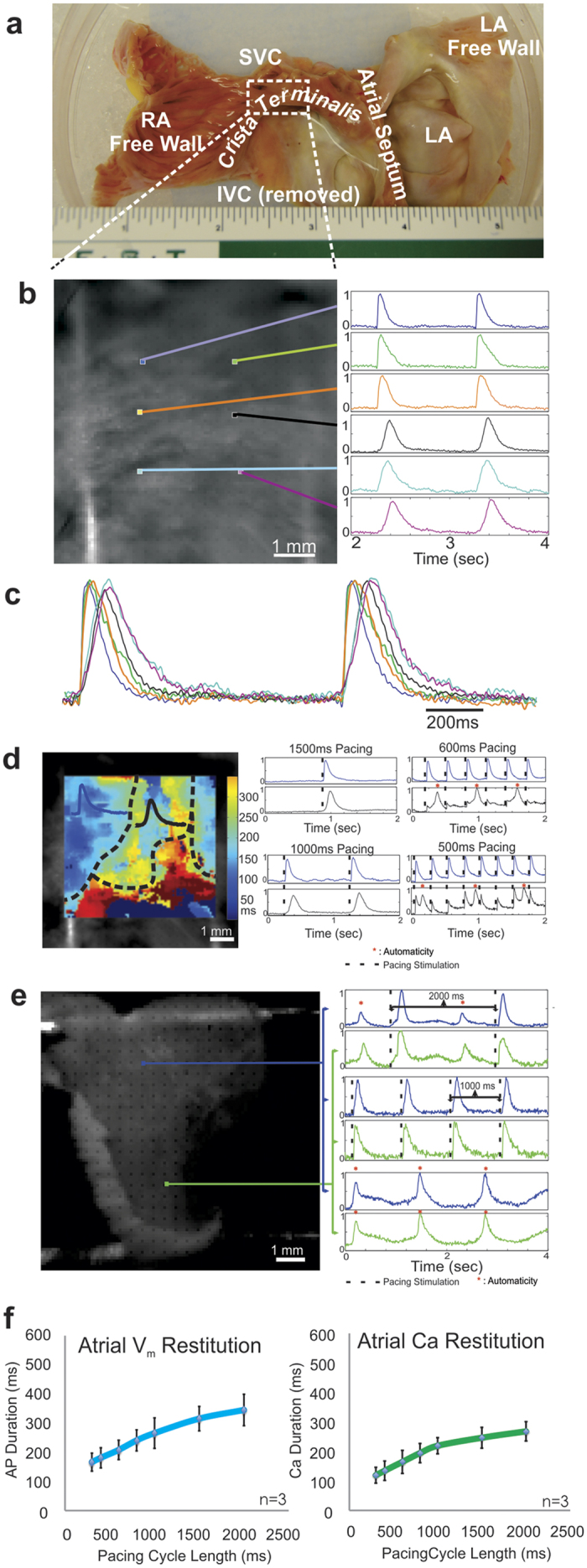
Measurement of Action Potentials and Calcium Handling in Human Atrial Slices. Action potential (AP) and calcium transient (CaT) can be measured in the human atria utilizing the slice model. (**a**) Whole atrial preparation prior to sectioning. Boxed region of the *crista terminalis* bordering the sinus node is shown in panel b. (**b**) Representative CaT recorded from various regions of an atrial slice. (**c**) CaT recorded from distinct atrial regions demonstrates heterogeneity in the CaT morphology. Faster Ca release and uptake (blue, green, and orange traces) were observed in regions of contractile atrial myocardium, while slower CaT upstroke and recovery (black, cyan, and purple traces) were recorded in the sinus node area. (**d**) Pacing at normal heart rates (1 Hz) resulted in complete CaT capture of both atrial and SAN regions, while at higher pacing frequencies (2 Hz) regional conduction block was observed only in the sinus node region due to the longer effective refractory period. (**e**) Pacing atrial slices containing the sinus node cells at various frequencies (0.5 Hz upper panels, 1 Hz middle panels, and no pacing lower panels) demonstrates normal sinus node activity with both spontaneous and captured AP. (**f**) Restitution curves of AP duration and CaT in atrial myocardium demonstrating shorter AP and Ca durations when compared with ventricular slices.

One of the critical advantages of the cardiac slice model is the ability to study any region of the heart regardless of availability of a branch of coronary artery required for perfusion in wedge preparations and/or size of the tissue sample. Previously, coronary perfusion was required to study human atrial and SA or AV nodal tissues^31^,^32^. Tissue slices can be sustained by superfusion as their thickness is below diffusion limit of oxygen, ~300–400 μm. Figure 4 illustrates our ability to optically map either AP or CaT in superfused atrial and SA nodal slices. A slice from the CT region bordering SA node is illustrated in Fig. 4a. In contrast to the ventricular optical AP and CaT, which are relatively homogeneous within each slice, optical CaT from the CT region consisting of both SA nodal and working atrial tissue exhibits significant heterogeneity in AP and CaT morphology and duration (Fig. 4b,c). Two distinctly different upstroke durations and morphologies of CaT were observed: SA nodal cells exhibited slow upstrokes and long duration of CaT (black, cyan, and purple), while the working atrial myocardium exhibited faster upstrokes and shorter CaT duration (blue, green, and orange). Furthermore, restitution pacing revealed heterogeneous effective refractory periods. At long pacing cycle length, we observed 1:1 capture in all regions. As pacing cycle length decreased, SA nodal tissue first lost 1:1 capture and then exhibited automaticity at ~1 Hz, which corresponds to a normal heart rate (Fig. 4d, black tracing). Simultaneously, the atrial muscle regions maintained 1:1 response as expected (Fig. 4d, blue trace). We also determined the inherent automaticity of slices from the SA node. Figure 4e illustrates optical AP recordings recorded during pacing at long cycle length of 2000 ms (30 beats per minute), where we observe both capture of paced beats as well beats originating from competing SA node firing once a second. With no stimulation, spontaneous SA nodal automaticity was observed (Fig. 4e). In contrast, ventricular and atrial muscle slices lacking the SA nodal region are electrically quiescent (Supplemental Fig. 1). We also quantified restitution properties in donor atrial muscle tissue for both AP and CaT (Fig. 4f). At 1 Hz stimulation, APD was 234 ± 16 ms (n = 3) and CaT duration was 219 ± 11 ms (n = 3). These atrial durations were significantly shorter than donor left ventricular APD and CaT duration (p = 0.0032, p = 0.002, *two tailed t-test assuming unequal variances*), in accordance with previous published APD data from coronary perfused preparations^12^,^33^. To our knowledge, this represents the first report of CaT data from both SA node and atria of the human heart.

### Novel adult human model to study chronic remodeling via drug stimulation

As demonstrated in Fig. 2, cardiac slices have a normal physiologic response to β-adrenergic stimulation. Yang *et al*. have recently shown the importance of also considering long-term drug responses when assessing cardiac drug efficacy and toxicity^34^. Using fresh and cultured ventricular slices, we developed a model of human cardiac remodeling under extended α_1_-adrenergic stimulation using 3 μM phenylephrine, an active compound of commonly used nasal spray.

**Figure 5:**
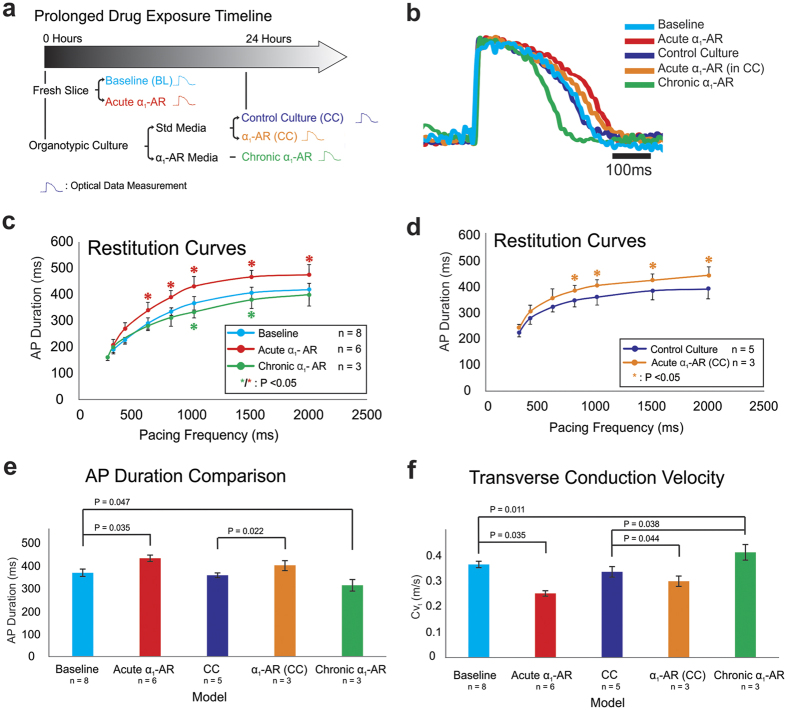
Application of the Slice Model System in Acute Versus Chronic Drug Testing. An acute versus chronic drug model is developed utilizing donor slices from the human ventricle. (**a**) A simplified timeline of the experimental protocol is shown. Various color corresponds to condition where optical measurement is taken. (**b**) Representative optical action potentials from control untreated ventricular slices (turquoise), slices treated acutely with α_1_-AR stimulation (15 min, red), control cultured slices (24 Hrs, blue), acutely treated slice after control culture (15 min, orange) and chronic α_1_-AR treatment (24 Hrs, green). (**c,d**) Restitution curves taken at each condition. Acute α_1_-AR stimulation both during baseline and control culture upshifted the restitution curve significantly while chronic α_1_-AR stimulation downshifted restitution (colored asterisk indicate significant difference of corresponding colored AP duration compared to baseline). (**e**) Statistical difference is calculated for AP duration between each condition at 1 Hz pacing frequency. Significant difference is observed whenever α_1_-AR stimulation is applied both acutely and chronically. Acute α_1_-AR stimulation increased AP duration, while chronic stimulation decreased AP duration. (**f**) Conversely, at 1 Hz pacing frequency, acute α_1_-AR stimulation significantly decreased transverse conduction velocity, albeit more prominently in fresh slices. However, chronic α_1_-AR stimulation increased transverse conduction velocity.

Figure 5a illustrates the timeline of the study. Figure 5b shows representative APD changes from baseline under different conditions. Importantly, APs at baseline recorded in fresh slices were no different from APs after culture in the absence of phenylephrine (cyan versus blue AP traces). Short-term application of phenylephrine in both fresh and cultured slices (red and purple AP traces, respectively), results in similar APD increases, as expected. In contrast, chronic α_1_-stimulation decreases APD (green AP trace). These changes in repolarization were further quantified through the restitution protocol (Fig. 5c,d), and individual values for APD and CV at 1 second pacing cycling length are plotted (Fig. 5e,f). Short-term α_1_-stimulation of fresh and cultured slices shifted the restitution curves significantly upwards, while chronic α_1_-stimulation shifted the restitution curve downwards. Given that the upward shift in the restitution curve in response to short-term α_1_-stimulation is similar in both acute and cultured slices (Fig. 5c,d, *one-way ANOVA with post-hoc Tukey HSD for each cycle length*), this suggests that cultured slices maintain normal physiologic responses.

To illustrate these differential changes we present quantification of APD recorded during pacing at 1 Hz. Fresh slices exhibited an APD of 368 ± 9 ms (n = 8) at baseline, while short-term α_1_-stimulation lengthened APD to 432 ± 9 ms (n = 6, Fig. 5e, p = 0.035, *one-way ANOVA with post-hoc Tukey HSD*). Slices cultured without α_1_-agonist exhibited APD of 357 ± 6 ms (n = 5), which increased to 401 ± 12 ms (n = 3) after short-term α_1_-stimulation (p = 0.022), similar to the increase in fresh slices. Interestingly, 24-hour chronic α_1_-stimulation, decreases the APD to 313 ± 20 ms (n = 3, p = 0.0047). These quantitative changes in APD lengthening in response to short-term α_1_-stimulation are similar to that observed previously in animal models^35^,^36^,^37^. However, our studies also reveal that prolonged α_1_-stimulation produces an opposite response in the APD, which has never been reported previously. Thus, we revealed potentially QT-toxic human effects of a commonly used nasal spray.

In order to accurately measure transverse conduction velocity, we used point stimulation of the slice instead of field stimulation in this experiment. Representative slice activation maps are shown in Supplemental Fig. 2. We find that CV also changes differentially with varying lengths of α_1_-stimulation (Fig. 5f). At 1 Hz pacing, fresh baseline slices exhibited transverse conduction velocity (CV_t_) of 0.37 ± 0.01 m/s, but short-term α_1_-stimulation slowed CV_t_ to 0.25 ± 0.02 m/s (p = 0.035, *one-way ANOVA with post-hoc Tukey HSD*). Cultured control slices exhibit baseline CV_t_ of 0.34 ± 0.03 m/s, which decreases to 0.26 ± 0.02 m/s after short-term α_1_-stimulation (p = 0.044). In contrast, chronic α_1_-stimulation significantly accelerates CV_t_ to 0.42 ± 0.02 m/s when compared with fresh (p = 0.011) or chronic (p = 0.038) untreated slices.

### Adenoviral-mediated gene expression in adult human ventricular slices

**Figure 6:**
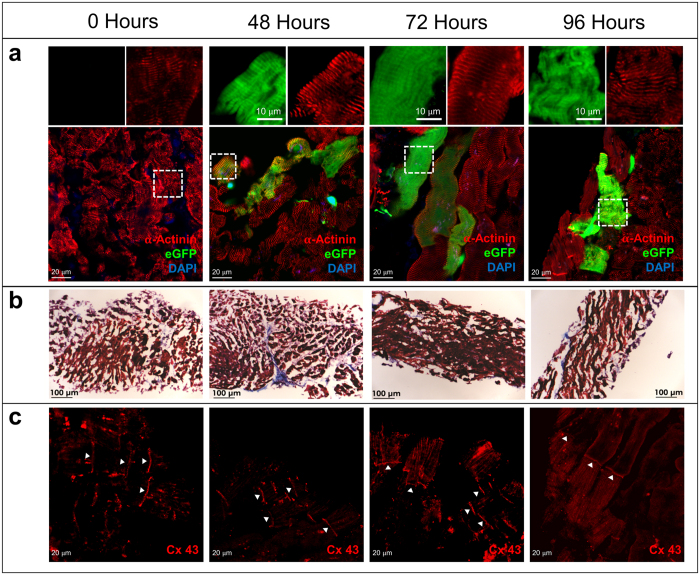
Adenoviral-mediated Gene Expression in Adult Human Ventricular Slices. Ventricular slices are transduced with adenovirus encoding eGFP shortly after harvesting. (**a**) Expression of eGFP in slices at time of harvest (0 Hours), and after 48, 72, and 96 Hours in culture. The lower panels show merged green (GFP), red (α-actinin) and blue (DAPI, nuclei) channels. Boxed regions are magnified in the top panels to show green (left) and red (right) channels separately. After 48 Hours, eGFP expression is robust in cardiomyocytes, as well as in non-myocytes, and eGFP expression persists throughout 96 Hours in culture. α-actinin staining demonstrates preservation of the sarcomeric structure throughout prolonged culture. (**b**) Masson’s Trichrome staining demonstrates preservation of ventricular slice structure throughout prolonged culture. (**c**) Immunohistochemical staining for Connexin 43, the main gap junction protein isoform expressed in human ventricular tissue, demonstrates maintenance of well-organized gap junctions at the intercalated disc throughout prolonged culture (white arrowheads).

Due to significant genetic and physiological differences between animal and human heart, putative therapies developed in animal models could fail in humans, or may exhibit unanticipated adverse effects in clinical trials. The ability to express exogenous genes within adult human myocardium could improve the understanding of human physiology and enable rapid screening and translation of putative therapies from the T0 to clinical trial stage. Specifically, the potential for cardiac slices to serve as an *in vitro* model of the adult human heart, both healthy and failing, may accelerate the development of gene therapy approaches. Here we demonstrate initial proof of concept that cardiac slices can be virally transduced and express an exogenous protein using adenovirus. In these studies, we extend the slice culture time to 96 hours to allow sufficient time for expression of virally-encoded eGFP and determine whether expression is maintained throughout culture. Adenoviral transduction shortly after harvesting slices resulted in expression of virally-encoded eGFP before 24 hrs (not shown), with maintenance of eGFP expression at 48–96 hrs (Fig. 6a). Slices were co-stained with an antibody recognizing the sarcomeric protein α-actinin, demonstrating localization of eGFP within myocytes, as well as eGFP expression within non-myocytes. The sarcomeric structure (Fig. 6a) and general tissue architecture (Fig. 6b) remains relatively preserved throughout culture. Immunohistochemical staining for Connexin 43 demonstrates maintenance of well-organized gap junctions at the intercalated disc during 96 hour culture (Fig. 6c). However, it is important to note that without electrical or mechanical loading, sarcomeric structure and gap junction alignment is likely to remodel.

## Discussion

By adapting and optimizing existing organotypic slice protocols, we successfully procured healthy culture ready atrial and ventricular slices from donor and failing human hearts. Acute optical mapping provided functional characteristics of slices comparable to traditional intact preparations. Twenty to thirty slices can be prepared from a small cubic tissue sample for optical mapping, culture, and viral infection. This approach allows high throughput, high resolution, and high accuracy screening of putative therapies for cardiac efficacy and/or toxicity.

### Slice validation using optical mapping

Multi-electrode array (Multi-Channel System) have been the most popular tool in electrophysiology studies of cardiac slices. Drawbacks of this approach include relatively low resolution and ambiguity of field potential interpretation. We show the feasibility of using multi-parametric optical mapping in human cardiac slices. Optical mapping provides high spatial and temporal resolution along with the ability to measure various parameters: transmembrane potential, intracellular calcium, inner membrane potential in mitochondria, etc. Left ventricular AP and Calcium recorded in both donor and failing slices were comparable to those recorded in both isolated primary cells and left ventricular wedge. Due to well known transmural electrophysiological differences in the left ventricle, in this study we used slices from the sub-endocardium mostly from the same location, 3^rd^–8^th^ slice from endocardium.

### Platform for studying human atria

AP mapping of coronary perfused human atrial preparations have been previously published by our laboratory^31^. However, human atrial calcium dynamics remained unknown. Our previous human atrial experiments required intact global or regional coronary perfusion, which was extremely difficult to achieve, making such experiments challenging to perform^32^. In contrast, cardiac slices alleviate this problem, since they do not require coronary perfusion. Slices can be prepared from any viable atrial regions, including biopsies. Approximately 5–10 slices can be made from each piece of atrial tissue.

In some atrial slices, we observed automaticity suggesting that there is a primary or secondary pacemaker present in these slices. For example, we observed automaticity in slices sectioned from the SA nodal region. Similarly, this approach can be used for systematic mapping of secondary or pathological pacemakers throughout the atria. Furthermore, different cell types could also be observed through the morphological differences in APs and CaT. The shorter AP/CaT region represents the working myocytes, whereas the longer AP/CaT regions with slower upstroke represent the pacemaker cells. Additionally, restitution properties of the two regions are distinctly different. While at 1 Hz pacing both regions were captured 1:1, at higher frequency this is no longer the case: at 600 ms and 400 ms pacing cycle lengths we observed 2:1 and 3:1 capture in SA node, respectively.

### Comprehensive model for pre-clinical drug testing

Recent reports shed light on an important, but often neglected difference between acute and chronic effects of drugs on electrophysiological targets^34^,^38^,^39^. Using human cardiac slices, we show here that acute versus chronic application of phenylephrine results in an opposite effect on APD. Clearly, there is an important signaling pathway activated by phenylephrine during chronic application, which remains to be explored in future studies. Isolated human primary cells and human coronary perfused preparations have a short lifetime, precluding long-term studies involving proliferative signaling. Animal models and human iPSC-derived cardiomyocytes could be used for chronic remodeling studies, but they offer limited recapitulation of adult human physiology. Thus, organotypic human cardiac slice offers a unique clinically relevant model for both acute and chronic human testing *ex vivo*.

### Adenoviral-mediated expression of eGFP

Gene therapy/editing has been advocated for numerous diseases. However, examples of successful clinical translation in the cardiovascular field are relatively few. We used eGFP as a proof of concept to suggest that organotypic human heart slice could be used to test the effects of exogenous genes expression in pre-clinical validation studies prior to embarking on costly human clinical trials. For example, an ideal viral vector would be available at high titers, have limited pre-formed immunity, and evade a host immune response to prevent dose-related toxicities. Adeno-associated virus (AAV) is currently being tested in clinical trials for myocardial gene delivery. Despite many useful properties of AAV, including prolonged *in vivo* expression and minimal immune response, significant limitations include difficulty producing sufficiently high titers, its restricted packaging capacity, and slow kinetics of transgene expression, which limits many applications. On this basis, several groups are adapting adenovirus (Ad) for cardiovascular gene therapy applications^40^,^41^,^42^,^43^. In this regard, two key facets of the Ad vector currently limiting its fullest utility are its dependence on the coxsackievirus and adenovirus receptor (CAR) for infectivity, and host anti-vector immune response. Strategies to confer Ad5 vectors CAR-independent myocardial tropism and evade a host immune response are underway, as previously described in other organ systems, which may expand the limits of gene packaging capability and enable the use of cell-type specific and inducible cardiac promoters for therapeutic applications^44^.

### Pre-clinical platform for novel therapies

Organotypic culture is an effective tool for cell therapies of diseases in central nervous system^7^. Similarly, adult human atrial and ventricular slices provide a versatile and potentially high throughput model for pre-clinical trials of novel therapeutic strategies. Acute administration of β-adenergic stimulation showed expected response on the APD and CV. Unexpectedly, acute versus chronic α_1_-adrenergic stimulation had opposite effects on APD and CV. This platform offers a unique adult human-specific platform for studying both acute (i.e. flight-or-flight) and chronic (i.e. proliferative) signaling at the tissue level. Various putative therapies could be tested in such platform, including pharmacological therapy, virally-medicated gene therapy, gene editing, cell therapy, tissue engineering, microRNA, gene editing, and device therapy.

## Additional Information

**How to cite this article**: Kang, C. *et al*. Human Organotypic Cultured Cardiac Slices: New Platform For High Throughput Preclinical Human Trials. *Sci. Rep.* **6**, 28798; doi: 10.1038/srep28798 (2016).

## Supplementary Material

Supplementary Information
