# Supplementary material for: Human Organotypic Cultured Cardiac Slices: New Platform For High Throughput Preclinical Human Trials
Source: Sci Rep. 2016 Jun 30;6:28798. doi: 10.1038/srep28798 (PMC4928074; doi:10.1038/srep28798)
Supplement: Supplementary Information [file srep28798-s1.pdf]

Human Organotypic Cultured Cardiac Slices: New Platform For High Throughput Preclinical Human Trials

Kang C, Qiao Y, Li G, Baechle K, Camelliti P, Rentschler S, Efimov IR

## Supplementary Table and Figures

| Preparation | Pro | Con |
|---|---|---|
| Isolated Primary Cells | - Only model for ion channels currents | - Absence of cell to cell coupling<br>- Chunk isolation process alters electrophysiology |
| iPSC- Derived Cardiomyocytes (Monolayers and Tissue Constructs) | - Highest throughput for therapy screening<br>- Genetic manipulation<br>- Patient specific sampling<br>- Unlimited tissue supply | - Unable to fully replicate adult tissue characteristics currently<br>- Cell alignment and cell coupling is disorganized |
| Coronary Perfused Intact Tissue | - Large Scale electrophysiology<br>- Tissue conduction properties<br>- Full scaled arrhythmia and defibrillation study | - Requires Intact coronary system<br>- Few preparations per heart<br>- Unable to be cultured |
| Tissue Slices | - Tissue level electrophysiology from anywhere on the heart<br>- Viable in organotypic culture for chronic studies<br>- Genetic manipulation | - Lower optical Signal-to-noise ratio compared to wedge preparations<br>- Cannot study true ionic currents<br>- Non-patient specific |

**Supplemental Table 1. Comparison between *ex-vivo* human cardiac models**

Pros and cons of each established and currently developing modeling for human *ex-vivo* cardiac study.

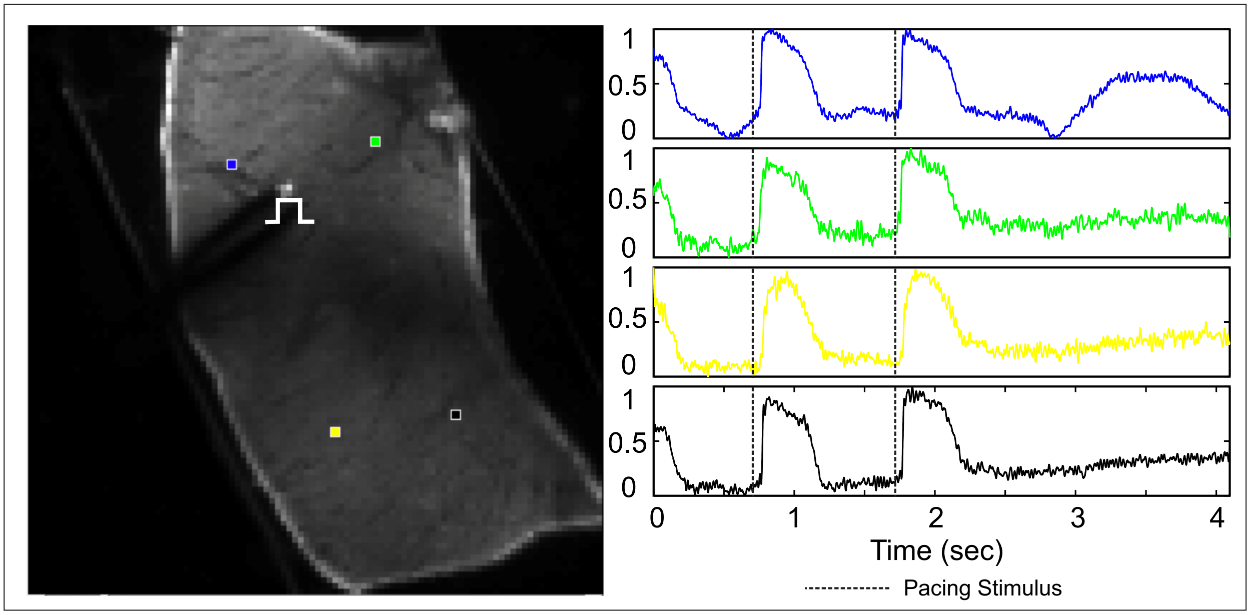


**Supplemental Figure 1. Quiescent Left Ventricular Slices**

Ventricular slices from donor tissue do not exhibit automaticity. Action potential can only be observed and recorded during electrical stimulation.

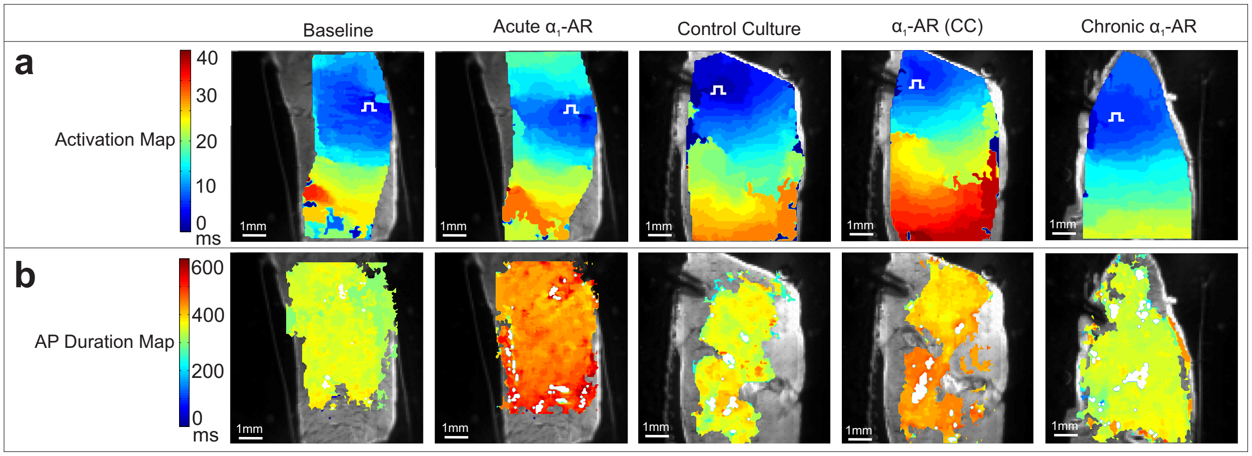


**Supplemental Figure 2. Activation and AP Duration maps of $\alpha_1$-AR stimulation**

Ventricular slices from donor hearts are treated either acutely (fresh and after control culture) or chronically. (a) Activation map of slices at each condition under 1 Hz pacing. Activation appears slower under acute $\alpha_1$-AR stimulation and faster under chronic $\alpha_1$-AR stimulation. (b) Representative AP duration maps of slices at each condition under 1 Hz pacing. Acute $\alpha_1$-AR stimulation clearly increased AP duration, while chronic $\alpha_1$-AR stimulation had the reverse effect.
